# Targeted Desorption Electrospray Ionization Mass Spectrometry Imaging for Drug Distribution, Toxicity, and Tissue Classification Studies

**DOI:** 10.3390/metabo13030377

**Published:** 2023-03-03

**Authors:** Andreas Dannhorn, Maria Luisa Doria, James McKenzie, Paolo Inglese, John G. Swales, Gregory Hamm, Nicole Strittmatter, Gareth Maglennon, Sadaf Ghaem-Maghami, Richard J. A. Goodwin, Zoltan Takats

**Affiliations:** 1 Department of Metabolism, Digestion and Reproduction, Faculty of Medicine, Imperial College London, London SW7 2AZ, UK; 2 Imaging and Data Analytics, Clinical Pharmacology and Safety Sciences, R&D, AstraZeneca, Cambridge CB4 0WG, UK; 3 Pathology, Clinical Pharmacology and Safety Sciences, R&D, AstraZeneca, Cambridge CB4 0WG, UK; 4 Institute of Infection, Immunity and Inflammation, College of Medical, Veterinary and Life Sciences, University of Glasgow, Glasgow G12 8QQ, UK

**Keywords:** DESI, MSI, mass spectrometry imaging, MALDI, TQ

## Abstract

With increased use of mass spectrometry imaging (MSI) in support of pharmaceutical research and development, there are opportunities to develop analytical pipelines that incorporate exploratory high-performance analysis with higher capacity and faster targeted MSI. Therefore, to enable faster MSI data acquisition we present analyte-targeted desorption electrospray ionization–mass spectrometry imaging (DESI-MSI) utilizing a triple-quadrupole (TQ) mass analyzer. The evaluated platform configuration provided superior sensitivity compared to a conventional time-of-flight (TOF) mass analyzer and thus holds the potential to generate data applicable to pharmaceutical research and development. The platform was successfully operated with sampling rates up to 10 scans/s, comparing positively to the 1 scan/s commonly used on comparable DESI-TOF setups. The higher scan rate enabled investigation of the desorption/ionization processes of endogenous lipid species such as phosphatidylcholines and a co-administered cassette of four orally dosed drugs—erlotininb, moxifloxacin, olanzapine, and terfenadine. This was used to enable understanding of the impact of the desorption/ionization processes in order to optimize the operational parameters, resulting in improved compound coverage for olanzapine and the main olanzapine metabolite, hydroxy-olanzapine, in brain tissue sections compared to DESI-TOF analysis or matrix-assisted laser desorption/ionization (MALDI) platforms. The approach allowed reducing the amount of recorded information, thus reducing the size of datasets from up to 150 GB per experiment down to several hundred MB. The improved performance was demonstrated in case studies investigating the suitability of this approach for mapping drug distribution, spatially resolved profiling of drug-induced nephrotoxicity, and molecular–histological tissue classification of ovarian tumors specimens.

## 1. Introduction

The majority of mass spectrometry imaging (MSI) studies employ non-scanning technology such as time-of-flight (TOF) [1,2], Orbitrap [3,4], or Fourier-transformation ion cyclotron resonance (FTICR)-based [5,6] mass analyzers to improve duty cycle and overall sensitivity. These experiments produce large datasets containing comprehensive information about the spatial distribution of thousands of chemical constituents. These constituents may include metabolic components, structural lipids, peptides, oligosaccharides, and proteins as well as various xenobiotics and their metabolites. The advantage of these large datasets is that they carry broad information about the overall status of the tissue and this information can further be interrogated to understand the underlying biochemical interactions. However, in most cases, the significant majority of the obtained information is not utilized, therefore monitoring of relevant features would be sufficient. Triple quadrupoles (TQs) operated in multiple reaction monitoring (MRM) mode are widely used for quantification purposes, taking advantage of their stable signal over a broad dynamic range. At the same time, TQs operated in MRM mode show superior sensitivity and comparable specificity compared to high-resolution mass analyzers. These advantages make TQs highly suitable and sufficiently robust for MSI analysis. Furthermore, MRM-based data acquired on a TQ mass spectrometer can increase throughput whilst reducing the amount of data to the required minimum, reducing costs for data analysis and storage. Within the AstraZeneca laboratory, we acquired routinely ~25 TB of MSI data per year using imaging setups with high mass resolving power. Using a targeted approach for studies where only a limited amount of the collected information is utilized, e.g., to evaluate drug disposition and distribution, this amount could potentially be reduced to several hundred GB. The reduced data could fit onto a single PC hard drive, limiting the need for expensive server-based or cloud storage arrangements whilst increasing data accessibility as the data can be housed and processed locally.

MSI-based drug metabolism and pharmacokinetics (DMPK) studies are part of the early phase drug development process and predominantly evaluate the distribution of drugs and their metabolites within distinct tissues or whole-body sections. The utility to monitor only selected features by MSI for DMPK studies were previously demonstrated by coupling of a matrix-assisted laser desorption/ionization (MALDI) source with a Q-trap mass spectrometer operated in MRM mode. Among others, this setup was applied to map the distribution of moxifloxacin in tuberculosis-infected rabbit lungs [7].

Previous reports demonstrated the possibility of urinary screening for illicit drugs and quantitation of pharmaceuticals in plasma by coupling desorption electrospray ionization (DESI) to a TQ operated in MRM mode [8,9,10]. Improved sensitivity through targeted analysis of pharmaceuticals was demonstrated investigating the suitability of DESI-MSI for drug disposition studies [10] and a recent report outlines the ability to use a DESI-TQ setup for targeted analysis of pharmaceuticals in tissues [11]. While these reports demonstrated improved sensitivity through targeted data acquisition, the implications of a DESI-TQ setup on scan speed, data quality, parameter optimization, and applications beyond the imaging of pharmaceuticals have not been explored yet.

In the current study, we demonstrate the suitability of DESI performed on a TQ for general MSI analysis. Targeted data acquisition was used to determine the desorption/ionization kinetics of compounds of interest in case of the given experimental setup. The resulting method was utilized for the elucidation of drug distribution in tissue sections, to monitor relevant biochemical pathways in a toxicology study, and for robust tissue classification and determination of tumor margins.

Such tissue analysis encompasses a significant percentage of MSI analytical capacity in an established MSI lab.

## 2. Materials and Methods

### 2.1. Chemicals

Analytical grade acetonitrile and water were obtained from Fisher Scientific (Loughborough, Leicestershire, UK). Isopentane, methanol, and acetic acid were obtained from Sigma-Aldrich (Poole, Dorset, UK). Test compounds for dosing were obtained in house from AstraZeneca compound management group (Macclesfield, Cheshire, UK), with the exception of moxifloxacin which was purchased from Sigma-Aldrich (Poole, Dorset, UK). Analytical standards of the drugs were obtained from Sigma-Aldrich (Poole, Dorset, UK), with the exception of erlotinib and Polymyxin B1 which were obtained from Cambridge Bioscience (Cambridge, UK). MALDI-MS grade 2,5-dihydroxybenzoic acid (DHB) was purchased from Sigma-Aldrich (Poole, Dorset, UK).

### 2.2. Tissue Processing for MSI

Tissues were cut into cryo-sections at a thickness of 10 μm and thaw-mounted onto SuperFrost microscope slides (Thermo Fisher Scientific Inc., Waltham, MA, USA) for DESI-MSI experiments or indium tin oxide (ITO)-coated MALDI target slides (Bruker, Bremen, Germany). For the drug distribution and toxicity studies, sections were taken where possible at approximately equal depth from all organs to allow visualization of similar structures between samples. Slides with mounted tissue sections were stored at −80 °C until analysis. Where applicable, organ tissue sections from dosed and vehicle control animals were mounted adjacent on the same slide and were analyzed in one experiment to limit the risk of any observed variation in relative abundance as a result in loss of analyzer sensitivity during the experiment.

### 2.3. Drug Distribution Studies

#### 2.3.1. Sample Collection

Adult male Han Wistar rats (approximate weight 260 g) were obtained from Charles River Laboratories (Margate, Kent, UK) and were acclimatized on site for a minimum of 3 days prior to dosing. Compounds were formulated in 5% dimethyl sulfoxide/95% (30% *w*/*v* Captisol in water) and administered via oral gavage. Control animals were dosed with the vehicle via the same administration route. The samples used were from a previously reported study exploring cassette-dosing as a tool to increase throughput in DMPK studies [12]. The animals were cassette-dosed with moxifloxacin, olanzapine, erlotinib, and terfenadine at 25, 10, 10, and 25 mg/kg, respectively. Animals were euthanized either 2 or 6 h post dose. All tissue dissection was performed by trained AstraZeneca staff (project license 40/3484, procedure number 10). Tissues (brain and liver) were snap-frozen in isopentane on dry ice, all subsequent transfer of tissues was carried out on dry ice, and samples were stored at −80 °C until tissue processing.

#### 2.3.2. Metabolite Identification

Single tissue sections were transferred into extraction tubes and zirconia beads and 0.5 mL of ice-cold methanol were added. To break up the tissue structure, the tubes were vortexed for 1 min and subsequently transferred for 5 min in an ultrasonic bath. The suspension was centrifuged for 10 min at 12,000 rpm under refrigeration and the supernatant was transferred into a new extraction tube and stored on ice. The remains were twice re-suspended in 0.5 mL ice-cold methanol, mixed in the vortex, centrifuged, and the supernatant pooled with the previous supernatant of the sample. Pooled supernatants were dried under nitrogen and reconstituted in 150 μL 25%/75% (*v*/*v*) methanol/water. The samples were again centrifuged under refrigeration for 20 min at 12,000 rpm to ensure quantitative removal of all particles. The supernatant was transferred into 300 μL glass insert vials and stored at −80 °C until used. To identify the drug metabolites present in the different tissues, tissue extracts were analyzed by LC-MS. Separation of the drugs and their metabolites was carried out on an UPLC system (Waters Acquity System, Manchester, UK) equipped with a BEH C18 column with the following dimensions: 100 mm × 2.1 mm i.d., 1.7 μm particle size (Waters, Manchester, UK). The column was heated to 55 °C and operated with a mobile phase flow rate of 0.5 mL/min. The eluent A was 0.1% aqueous acetic acid and the eluent B was 0.1% acetic acid in methanol. Next, 5 μL were injected per sample and separated with the following linear gradient: initially 95% A/5% B, T = 0.5 min 95% A/5% B, T = 8.00 min 10% A/90% B, T = 8.01 min 0% A/100% B, T = 8.50 min 0% A/100% B, T = 8.51 min 95% A/5% B, T = 9.00 min 95% A/5% B. The mass spectrometric analysis was performed with a Xevo G2-XS Q-TOF (Waters, Manchester, UK) operated in positive ToF-MS mode over a mass range from *m*/*z* 50 to 1200. Additionally, a data-independent MS/MS function (MS^e^) function was acquired to obtain MS/MS spectra for the compounds eluting from the column. The collision energy for the MS^e^ acquisition was ramped from 25 to 40 V to obtain a broad variety of fragment ions.

#### 2.3.3. DESI-MSI

DESI-MSI experiments were performed either on a Xevo G2-XS Q-TOF or a Xevo TQ-S (Waters, Wilmslow, UK) equipped with a 2D sampling stage (Prosolia Inc., Indianapolis, IN, USA) and a custom-built inlet capillary heated to 500 °C. The nominal pixel size was set to 50 × 50 μm. The Q-TOF setup was equipped with a commercially available DESI spray head (Waters, Manchester, UK) [13]. The imaging regions were selected, and the resulting data processed in the High-Definition Imaging software (version 1.4) (Waters, Manchester, UK). Data were acquired in positive ion mode over a mass range of *m*/*z* 50 to 1200 for TOF-MS mode and *m*/*z* 50 to 750 for TOF-MS/MS mode. The collision energies for MS/MS acquisitions were manually optimized on the Q-TOF. The acquisition speed was set to 1 scan/s.

The TQ setup was equipped with a home-built sprayer. The stage movement was controlled through Omni Spray 2D (Prosolia Inc., Indianapolis, IN, USA). The scan speed was set to 10 scans/s. Positive ion mode MRM transitions were optimized for the drugs, their metabolites, and endogenous metabolites. The latter signals were used to establish the tissue outline. Drug standards were infused into the mass spectrometer through the commercial ESI source and MRM transitions were optimized by running the auto-optimization function in the operating MassLynx (version 4.1) software. MRM transitions for the drug metabolites were created based on their fragmentation pattern obtained from the MS^e^ function of the LC-MS based metabolite identification whilst transitions for endogenous metabolites were established based on the fragmentation pattern obtained by DESI-MS/MS analysis performed on a Q-TOF. The final transitions are given in Supplementary Table S1. The collision energies for both species were subsequently manually optimized on the TQ to achieve the highest MS response. Separate line scans were performed over the imaging area and the resulting data files were processed in an in-house developed toolbox operating in MATLAB environment. The different functions were relatively scaled and overlaid where applicable to allow visualization of the drugs and metabolites within the tissue section.

#### 2.3.4. MALDI-MSI

When removed from the −80 °C freezer, thaw-mounted tissue sections were immediately dried under a stream of nitrogen to prevent water condensation on the sample surface. The tissue was coated with DHB following a protocol previously optimized for these samples [12,14]. Briefly, a TM-Sprayer sample preparation system (HTX Technologies, LCC, Carrboro, NC, USA) was used to apply the MALDI matrix. DHB was dissolved in 50:50 *v*/*v* acetonitrile/water containing 0.1% TFA giving a final concentration of 37.5 mg/mL. The matrix solution was delivered with a flow rate of 80 μL/min and nebulized with a gas pressure of 10 psi. The nozzle temperature was set to 75 °C. A total of 8 passes of matrix application were performed in “criss-cross” pattern. MALDI imaging was performed using a rapifleX MALDI Tissuetyper (Bruker Daltonics, Bremen, Germany) system equipped with a smartbeam 3D laser. Data acquisition was controlled through Bruker’s Fleximaging software (Version 5.1). The resulting data were converted into imzML files and processed in msIQuant [15]. The pixel size was set to 50 × 50 μm and 400 laser shots were collected per pixel with a laser repetition rate of 5 kHz. The data were acquired in positive ion mode over a mass range from *m*/*z* 200 to 1000.

### 2.4. Toxicity Study of Polymyxin B Induced Kidney Injury

#### 2.4.1. Sample Collection

Adult male Han Wistar rats (approximate weight 260 g) were obtained from Charles River Laboratories (Raleigh, NC, USA) and were acclimatized on site for a minimum of 3 days prior to dosing. Compounds were administered through subcutaneous injection in the intrascapular region at 25 mg/kg/day for 3 days. Control animals were dosed with saline vehicle via the same administration route. All tissue dissection was performed by trained AstraZeneca staff (project license 40/3484, procedure number 10). Tissues were snap-frozen in 2-methylbutane on dry ice; all subsequent transfer of tissues was conducted on dry ice, and samples were stored at −80 °C until tissue processing.

#### 2.4.2. Mass Spectrometry Imaging

For DESI MSI experiments performed on the TQ, MRM transitions were optimized based on the identified fragmentation spectra of previously identified metabolites and are summarized in Table S2. Collision energies were manually optimized to achieve highest MS response for each compound. Subsequently, the entire sample set was re-analyzed. In each ion mode, a total of approx. 50 MRM transitions were acquired, resulting in a dwell time of 4 ms/transition and a total acquisition rate of 2 pixels/s. These settings allowed data acquisition with a balance of spatial resolution and sensitivity. Targeted imaging experiments were performed with a spatial resolution of 75 μm.

### 2.5. Tissue Classification Study

#### 2.5.1. Clinical Sample Collection

A proof-of-principle tissue classification study was performed on three high-grade serous ovarian carcinoma samples containing both tumor tissue and stroma tissue, and 2 healthy ovarian samples with healthy histological components. The collection of all samples for this study was approved by the institutional review board at Imperial College Healthcare National Health Service Trust (Tissue Bank sub-collection number GYN/HG/12/060). All patients provided informed consent to the use of their samples in this study, and all methods were performed according to institutional and ethical guidelines. All tissue samples were stored at −80 °C after collection.

#### 2.5.2. Mass Spectrometry Imaging

Classification experiments were performed as above on a Xevo TQ-S equipped with a 2D sampling stage (Prosolia Inc., Indianapolis, IN, USA). The setup was equipped with a commercially available DESI spray head (Waters, Manchester, UK) operated with a mixture of 95%/5% (*v*/*v*) methanol/water delivered with a flow rate of 2 μL/min. A custom-built inlet capillary heated to 500 °C was used for ion transfer from the sample surface into the mass spectrometer.

The collision energies for the targeted metabolites were optimized on sample tissues to achieve best MS response. Data were recorded with a spatial resolution of 100 μm with an acquisition rate of 1 scans/s to maintain clear spatial resolution of the fine tissue structures. Subsequently, all tissue sections were stained with hematoxylin and eosin (H&E) and underwent histological examination by a histopathologist. The MSI data were directly uploaded and processed in an in-house developed toolbox running in Matlab (Math-works, Natick, MA, USA).

All instrumental details and MRM transitions used are summarized in the supplementary information.

## 3. Results

### 3.1. Drug Distribution Study

**Figure 1:**
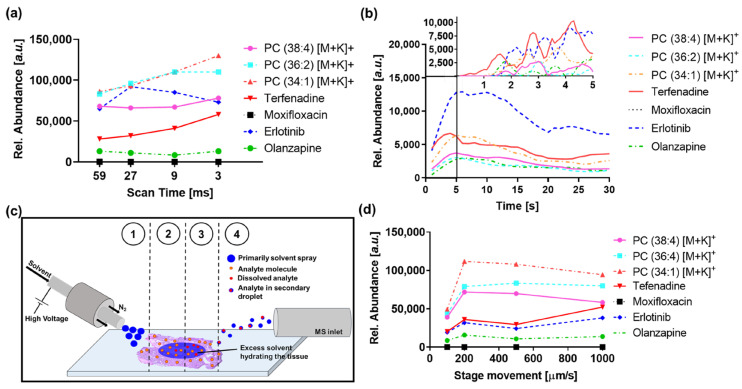
(**a**) Relative abundances of endogenous compounds and cassette-dosed drugs over dwell times of 59, 27, 9, and 3 ms/transition. PC-phosphatidylcholine. Data represent the mean of 3 randomly chosen ROIs per data-point. (**b**) Kinetics experiment monitoring the same transitions were performed with a dwell time of 59 ms resulting in a scan rate of 1 scans/s. The zoomed panel shows the static kinetics of the first 5 s acquired with a dwell time of 3 ms resulting in a scan rate of 10 scans/s. All data represent the mean of 3 independent experiments. (**c**) Schematic visualization of the steps involved in the desorption/ionization process: (1) Re-hydration of the tissue section through primarily charged electrospray solvent, (2) Accumulation of excess spray solvent in the tissue section and dissolution of analytes, (3) Analytes are increasingly dissolved in excess spray solvent, and (4) desorbed in secondary droplets which enter the mass spectrometer. For easier accessibility of the schematic not visualized is step (5), depletion of analytes from target tissue. (**d**) Desorption dynamics for the monitored compounds for stage movement of 100, 200, 500, and 1000 μm/s acquired at a constant dwell time of 9 ms resulting in a scan rate of 5 scans/s. Data represent the mean of 3 independent experiments.

After the optimization of the MRM transitions for the drugs and endogenous lipid species, the effects of dwell-time, desorption kinetics, and dynamics on achievable data quality were evaluated using the MRM transitions described in Table S4. Relative abundances of the four drugs and three endogenous lipids were compared at dwell times of 59, 27, 9, and 3 ms corresponding to acquisition rates of 1, 2, 5, and 10 pixel/s, respectively (Figure 1a). For all experiments, the stage movement was adapted to the scan speed and every spectrum represents 100 μm of scanned sample. For each dwell time, 25 consecutive line scans were performed. The raw files were processed in Matlab to extract the mean intensities. For each dwell time, the spectral information was exported as the mean of 36 pixels. For highly abundant endogenous lipids and terfenadine, the relative abundances increased with shorter dwell times, whilst the abundances for erlotinib and olanzapine showed no direct dependence on the dwell time. Only the low abundance moxifloxacin showed a dwell time-dependent drop in abundance. With a maximum of 190 counts at a dwell time of 59 ms and no clear corresponding ion image for the drug, moxifloxacin abundances are mainly below the limit of detection even on the longest dwell time tested. The ion abundance values reported by the instrument were normalized to the dwell time, thus the abundances of each analyte should be constant across all scan speeds. To explain the deviation from a linear relationship of signal response to dwell time, we evaluated the kinetics and dynamics of the DESI part, taking advantage of the fast scan rates of the setup. Desorption kinetics acquired under static conditions for the monitored drugs and lipids show an initial delay in detection when the spray is directed onto a tissue section, followed by an increase in signal intensity, plateauing, and subsequent decline (Figure 1b).

The distinctive phases can be correlated with the steps of the desorption/ionization process illustrated in Figure 1c: The initial delay is based on the required re-hydration of the tissue section and dissolution of the compounds in the spray solvent. With increasing excess spray solvent present on the tissue surface, the desorption/ionization becomes more efficient as more of the analytes are dissolved at a given time, resulting in signal increase with a slope defined by the dissolution kinetics of the compound in the spray solvent. The plateau marks the point for the process when dissolution kinetics becomes the rate-limiting step for the process. The final decline of the signal demonstrates exhaustion of the tissue section and depletion of analytes that can be desorbed/ionized. For an imaging application with a high scan rate, the initial steps are particularly significant as they most closely represent the conditions used for the imaging experiment. The desorption dynamics for the initial seconds of spray solvent reaching the tissue were monitored with a scan rate of 10 scans/s (Figure 1d). The compounds show different time onsets for the signal detection, with terfenadine, erlotinib, and olanzapine displaying the fastest desorption/ionization with first maxima observed between 1 to 2 s, whilst the structural lipids display a slower desorption/ionization with maxima observed between 2 to 3 s. Interestingly, all compounds show an oscillating pattern. Terfenadine and olanzapine tend to show maxima at similar times, whilst erlotinib anticorrelates and displays the highest response when olanzapine and terfenadine valley, suggesting potential charge competition/suppression. The abundance of compounds observed in DESI-MSI experiments is not just dependent on the desorption kinetics but also on the speed of stage movement. Additional experiments with constant scan rate and differential stage movement speed were performed to investigate the contribution of the dynamic stage movement to the detected signal. When increasing the stage movement speed from 100 μm/s to 200 μm/s, the detected signal for all compounds doubled as expected, as twice the amount of tissue was analyzed. However, when the stage movement was increased to 500 μm/s, the signal for most compounds dropped slightly, most likely due to a shift in the detection from the exhaustion phase into the plateau phase observed in the kinetics experiments. Further increase in the stage movement results in a further decrease in the monitored lipids whilst the signal detected for the drugs increases to a maximum, consistent with a shift into the dynamic front region of the kinetics experiments, the phase in which the desorption/ionization is dependent on the dissolution speed of the target compound. This behavior allows careful optimization of the setup for target analytes, especially when they show differential dissolution kinetics than the main tissue components.

These findings are overall in agreement with early reports on the desorption/ionization process of small molecules from tissue sections [10,16]. The parameter optimization for the DESI-TQ setup highlights the importance of the dissolution time for surface solids and thus sample stage movement speed, which ultimately drives the impact of scan speed on the MS response. The impact of the data acquisition speed on spectral composition should be independent of the mass analyzer used for the imaging experiment with the same fundamentals impacting instruments with short duty-cycles, such as instruments utilizing time-of-flight mass analyzer technology capable of reaching scan-speeds of 10 spectra/s or more.

**Figure 2:**
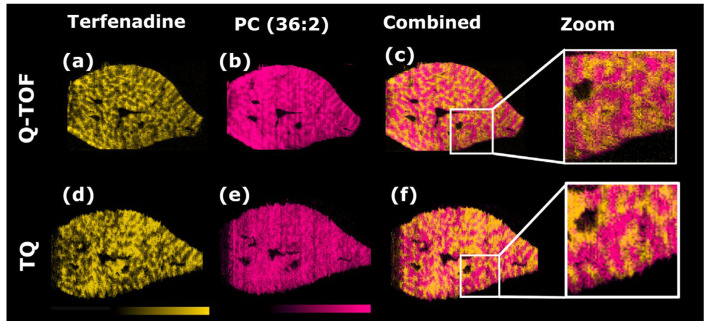
Ion images for terfenadine (**a**,**d**) and endogenous lipid PC(36:2) (**b**,**e**) obtained by DESI-MSI performed on a Xevo G2-XS or a Xevo TQ-S. (**c**,**f**) show the combined ion images of the drug and the endogenous lipid. The zoomed view displays the tissue edges. Images (**d**–**f**) were re-aligned as described in the (Supplementary Information Figure S1). The original, unprocessed images can be found in Figure S2.

The achievable image quality using optimized data acquisition parameters is displayed in Figure 2, which shows the distribution of terfenadine in rat liver sections 2 h post dose. The images obtained on the Q-TOF and TQ setup delivering fundamentally identical information by generating comparable distribution maps for terfenadine within the liver. Both imaging platforms detected terfenadine predominantly in the periportal zone (zone 1), distinctly separated from the endogenous lipid PC(36:2) highlighting centrilobular areas (zone 3).

Although both datasets were acquired at the same nominal spatial resolution of 50 μm, the DESI-TQ setup appears to exhibit lower feature resolution compared to the Q-TOF setup. The image distortion is introduced by an instrument-dependent de-synchronization between stage movement and the start of the data acquisition. The de-synchronization results in line-to-line jitter of a few pixels. The effect is more pronounced with increasing scan rates. The shifts were compensated in Figure 1 by re-aligning the individual line scans as described in the supplementary information. The original images are displayed in Figure S2.

Overall, the new setup allowed collection of data on the spatial distribution of all four dosed drugs and selected endogenous species could be acquired with high sensitivity and specificity. The resulting data file size, for a whole microscope slide holding three liver sections, was reduced from around 100 GB for the Q-TOF data to 873 MB for the 16 acquired MRM transitions on the TQ. Additionally, the data acquisition rate could be increased from 1 scan/s on the Q-TOF to 10 scans/s on the TQ.

**Figure 3:**
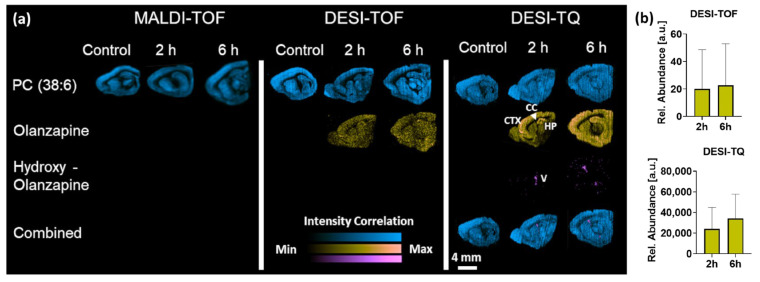
(**a**) Distribution of endogenous lipid PC(38:6), olanzapine, and its metabolite hydroxy-olanzapine in sagittal rat brain sections collected 2 and 6 h post dosing compared to a vehicle control. Distributions were determined by MALDI and DESI-MSI. DESI-MSI experiments were performed on a standard Q-TOF or a TQ-setup. All data were acquired with a spatial resolution of 50 μm CTX = frontal cortex, CC = corpus callosum, HP = hippocampus, V = ventricle. (**b**) Relative abundances of olanzapine as detected by the different instruments. Data presented as mean ± SD.

To evaluate the sensitivity of the new setup, we compared the detection level of olanzapine and its metabolite hydroxy-olanzapine in sagittal rat brain sections. The TQ setup was compared to the Q-TOF and a MALDI-TOF setup to evaluate the sensitivity and data quality. As expected, the MALDI setup creates images with high spatial resolution and sharp outlines of the tissue structures (Figure 3). However, even though the matrix application was previously optimized for the detection of olanzapine [12], neither olanzapine nor its hydroxy-metabolite could be detected within brain tissue sections.

The DESI-Q-TOF setup delivered similar sharp ion images for endogenous compounds acquired in full scan mode. Data acquired in MS/MS mode clearly show the presence of olanzapine within the tissue section, but based on the limited sensitivity, a clear identification of areas with drug accumulation is not possible. As for the MALDI setup, the sensitivity of the DESI-Q-TOF setup was not high enough to map the distribution of the hydroxylated metabolites within the tissue. The data acquired on the DESI-TQ setup reveal accumulation of olanzapine in the frontal cortex (CTX) and hippocampus (HP) whilst it could not be detected in the corpus callosum (CC). The improved sensitivity of the TQ setup was reflected in a more defined difference of the mean abundance detected for olanzapine between the 2 and 6 h timepoints and the overall reduced pixel-wise standard deviation due to a reduced number of pixels in which the drug was not detected (Figure 3b). The improved sensitivity also enabled the detection of the hydroxylated metabolites that were based on the same fragmentation pattern captured in the same MRM transition. The overlay with the endogenous lipid PC(38:6) locates the drug in the ventricle (V), where it most likely accumulates in the choroid plexus. The observed inability of hydroxy-olanzapine to penetrate the blood–brain barrier is consistent with previous reports [17].

The increased sensitivity of the DESI-TQ setup for olanzapine and its metabolites stand in contrast to previous work published by Lamont et al. who evaluated the LOD for two drug candidates on a similar instrument setup using liver tissue mimetics [18]. The use of such mimetic-based calibration standards is required for the reported quantitative MSI workflow and does enable to approximate the sensitivity of the different instrument setups delivering a quantitative readout. However, the approach does not fully recapitulate the desorption/ionization kinetics of intact tissues and does not inform on the effective imaging sensitivity of setups or on pixel-wise variability of the data, which ultimately drives the data quality. The here-presented work focused on compound detection and quality of the resulting imaging data.

### 3.2. Toxicity Study: Polymyxin B Induced Kidney Injury

The performance of the DESI-TQ setup was subsequently evaluated using samples from a toxicity study to evaluate applications of the setup beyond the imaging of drugs and their metabolites in tissues. Monitoring of endogenous metabolites informing of the state of a tissue and drug-induced toxicity findings in a high-throughput manner holds the potential to develop organ- or toxicity-specific assay panels that could be readily run to monitor metabolic changes associated with tissue homeostasis and damage. The resulting datasets would be easier to interpret due to the targeted data acquisition of previously identified and validated metabolites. This section will demonstrate an exemplified workflow on rat kidney sections from animals dosed with Polymyxin B1 (PMB) showing drug-induced acute kidney injury. The drug is known to accumulate in the proximal tubules located in the renal cortex [14], where it is actively taken up into epithelial cells via phagocytosis/pinocytosis. Upon merging with lysosomes, PMB is ionized due to the reduced pH in the resulting phagolysosomes and endosomes effectively trapped in these lysosomal fractions, disrupting lysosomal/endosomal function and preventing secretion into the tubule lumen. Trapping of PMB also leads to impaired lysosomal/endosomal function resulting in single cell necrosis of the epithelial tubule cells and drug-induced kidney injury [19,20].

Metabolites selected for targeted data acquisition in this work were selected from DESI-full scan experiments performed on a Q-Exactive (Thermo Scientific, Bremen, Germany) mass spectrometer. The full datasets were processed in MSiReader (v0.09) [21], where regions of interest (ROIs) were annotated for renal cortex, outer and inner medulla, and data extracted with 0.1 Da binning and subjected to univariate statistics (two-tailed t-test, *p* < 0.05) to identify significant metabolic changes between the control and dosed group. Features with significantly different abundances were identified by DESI-MS/MS analysis performed on the Q-Exactive mass spectrometer and the fragmentation pattern used to generate MRM transitions for the targeted imaging approach. The here-presented work focusses on some metabolites of interest from this dataset where the targeted image acquisition proved particularly interesting as some of the identified lipid species were found to be mixtures of structural isomers that could be resolved based on their fragmentation pattern, and simultaneously imaged. The MRM transitions monitored can be found in Table S5.

**Figure 4:**
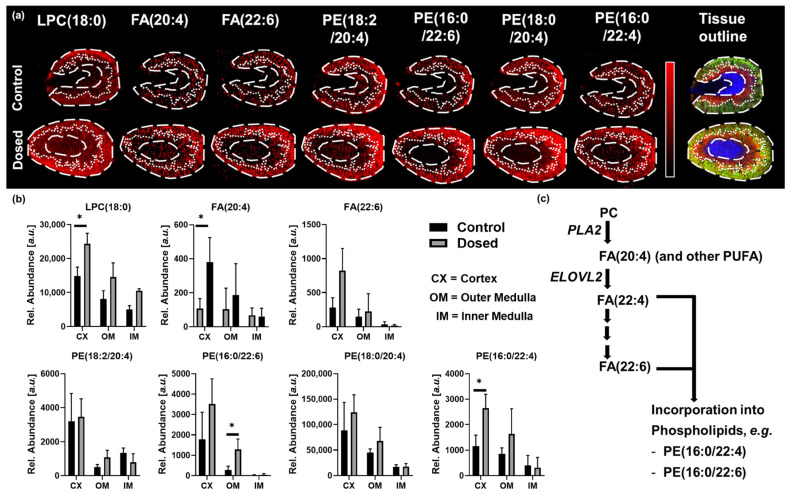
(**a**) Ion images for molecules associated with inflammation: LPC(18:0), fatty acid(20:4) (FA(20:4)), and fatty acid (22:6) (FA(22:6) and resolved pairs of the isobaric phospholipids PE(18:2/20:4) and PE(16:0/22:6), PE(18:0/20:4), and PE(16:0/22:4), respectively. The RGB image with the tissue outlines for inner medulla (blue), outer medulla (red), and cortex (green) for control and dosed sections. (**b**) shows the relative abundance of the metabolites highlighted in a. Data are presented as mean + standard deviation of 4 randomly chosen ROIs across cortex, outer and inner medulla. Statistical significance was determined via two-tailed, non-parametric test (Mann-Whitney); Significance level * = *p* < 0.05 (**c**) Metabolic pathway scheme for the detected metabolites.

Increased levels of arachidonic acid (*m*/*z* 303.23) and docosahexaenoic acid (DHA) (*m*/*z* 327.23) were detected in the kidney upon dosing with PMB (Figure 4a). Activation of Phospholipase A2 (PLA2) cleaves PC species containing poly-unsaturated fatty acids (PUFA) in sn-glycero-2-position, liberating the PUFA under formation of a remaining lyso-phosphatidylcholine (LPC). PUFAs are indicative for activation of the inflammation-regulating eicosanoid pathway [22]. LPCs are involved in tissue inflammation by promoting mononuclear cell infiltration into the tissue [23,24]. Adrenic acid is synthesized by elongation of arachidonic acid (FA 20:4) through elongation of very long-chain fatty acid elongase 2 (ELOVL2) enzymes on the biochemical pathway to synthesis of DHA. Synthesis of DHA requires further elongation to a C-24 fatty acid followed by dehydration and partial β-oxidation [25]. Targeting the fatty acid carboxylate fragments in negative ion mode allowed to distinguish between different phospholipids from the same class with the same chemical formula but different fatty acid chain compositions. This specificity allowed to distinguish between different isobaric phosphatidylethanolamines (PEs). Figure 4b shows the distribution of PE(18:2/20:4) and PE(16:0/22:6) detected as [M−H]^−^ at *m*/*z* 762.51. Both lipid species have a comparable distribution across the different tissue types, with PE(18:2/20:4) having a higher abundance in the inner medulla compared to its isomer.

Upon dosing with PMB, PE(18:2/20:4) showed comparable abundances for dosed and control animals whilst PE(16:0/22:4) showed a more than 2-fold increase (Figure 4b). A second pair of transitions acquired in the same experiment allowed us to distinguish between two species of PE(38:4) detected as [M−H]^−^ at *m*/*z* 766.54. The performed MS/MS experiments identified the biologically anticipated lipid PE(18:0/20:4) and also a lipid PE(16:0/22:4). Targeting of the fatty acid chains allowed to map the spatial distribution of both species to visualize and analyze their differential distribution (Figure 4a). As biologically expected, the lipid PE(18:0/20:4) shows overall higher abundance and a distinct difference between different tissue types of the specimen (Figure 4b). Whilst PE(16:0/22:4) has a comparable distribution pattern, it shows a lower abundance overall. Overactivated de-novo lipid biosynthesis could drain the fatty acid from the biochemical pathways resulting in the observed lipid species. The metabolic pathway scheme is illustrated in Figure 4c. However, the exact biological role of these phospholipids in this induced acute kidney injury model exceeds the scope of this methodology-focused work and requires further investigation.

We successfully used the setup to monitor biochemical pathways associated with inflammation, mononuclear cell infiltration, and general kidney function. Relevant pathways with altered abundances of the compounds involved were identified on a dosed subset of the sample collection. Spatially resolved targeted analysis allows visualization and analysis of compounds associated with biochemical pathways of interest.

### 3.3. Tissue Classification Study

The above-described ability to monitor metabolic changes associated with drug-induced tissue toxicity in a targeted way opens the potential to purely monitor the distribution of endogenous metabolites in tissues by applying a similar workflow. One of the application areas with demand for a high-throughput imaging platform is clinical tissue classification. Tissue classification studies are commonly performed on data obtained from mass spectrometry equipment with high mass-resolving power to gain insight into the molecular composition of tumors and the tumor microenvironment, intratumor heterogeneity, or understand pharmacotherapeutic effects. These untargeted datasets allow deep molecular profiling and study of biological processes in the analyzed tissues [26,27]. The data generated in such research studies could be utilized to provide molecular–histological insights during tumor and tumor margin detection in the clinic alongside histopathological evaluation. However, data generation and analysis are time consuming for such datasets and require expertise to operate the equipment and perform the data analysis in order to obtain accurate and insightful results. The complexity of untargeted data generation and mining limits the ability of routine clinical application where higher throughput is required to be incorporated in established workflows. The high scan rates, pre-defined data acquisition, and physical robustness of the evaluated DESI-TQ setup are ideally suited for seamless and automated integration into clinical workflows.

Key metabolites driving the classification in previously reported work were selected [28] to perform a proof-of-concept evaluation of targeted data acquisition for tissue classification purposes. The analysis was performed on 14phosphatidylcholine (PC) and sphingomyelin species in positive ion mode. Transitions were chosen using previously recorded MS/MS fragmentation data which can be found in Table S6. The sample content presented in this study is limited as it is a first proof-of-principle investigation to evaluate the feasibility to use the presented DESI-TQ platform for tissue classification. The purpose of this study was not to identify biochemical differences in a comprehensive clinical setting.

**Figure 5:**
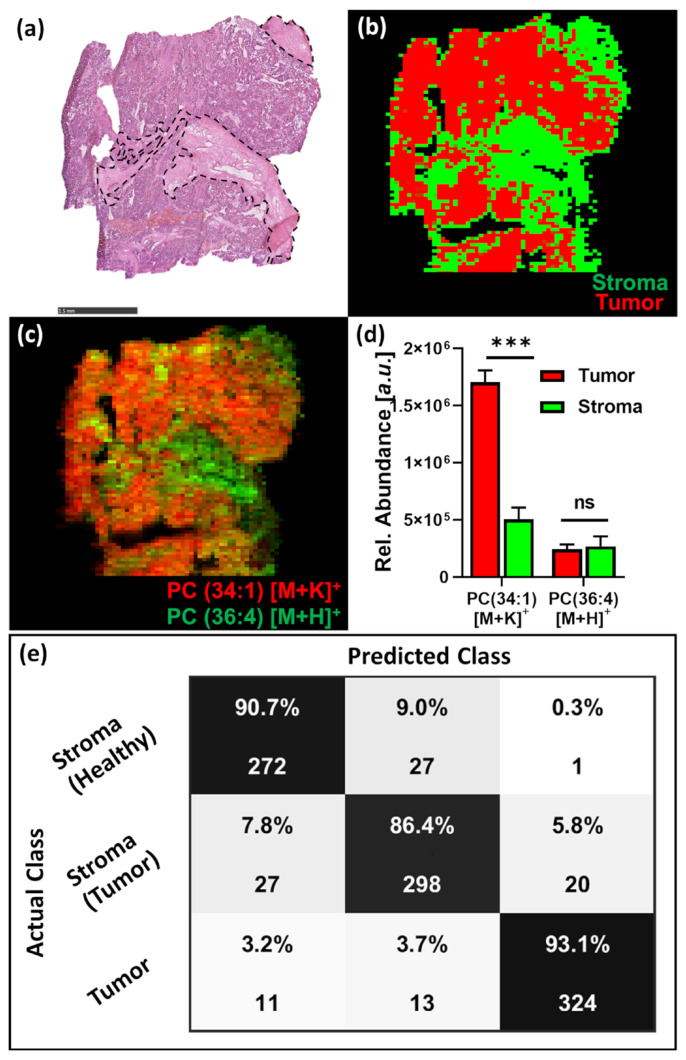
(**a**) Post-DESI-MSI H&E-stained tissue section of the ovarian tumor sample, scale bar = 2.5 mm. (**b**) MMC segmentation into tumor (red) and stroma (green), unclassified pixels are displayed as background (black). (**c**) Combined ion images for PC(34:1) and PC(36:4), and (**d**) relative abundances for these lipids in tumor and stroma tissue. (**e**) Ten-fold PLS random sub-set inter-sample cross-validation for 3 ovarian carcinoma samples and 2 healthy stroma samples. Bar charts represent the mean ± SD of annotated pixels. Statistical significance was determined via two-tailed, non-parametric test (Mann-Whitney); Significance level *** = *p* < 0.0001, ns = not significant.

The distinct differences in analyte abundance allow pixel-by-pixel classification of tumor and stroma containing ovarian carcinoma samples (Figure 5a) using supervised Linear Discriminant Analysis with Maximum Margin Criterion (MMC) based on manual annotation of pixels as either tumor or stroma, based on the post-MSI H&E-stained tissue sections. The analysis accurately segmented the tissues into tumor (red) and stroma (green) (Figure 5b) matching the H&E-stained section.

Pixels that could not be classified as either tumor or stroma were removed from the segmentation and considered as background pixels displayed in black. MMC 10-fold random sub-set cross-validation for the annotated pixel within the sample resulted in 100% accuracy. While the monitored lipid PC(36:4) showed a rather homogenous distribution overall with slight elevation in the tumor-associated stroma, the PC(34:1) showed increased abundances in the tumor regions (Figure 5c,d). The tissue outlines of the segmentation based on these two metabolites match the tissue outlines determined through MMC segmentation based on all acquired data and are in agreement with the histological evaluation of the section.

Partial least squares (PLS) 10-fold random subset cross-validation for annotated pixel of all samples could be performed with good accuracy for tumor vs. stroma tissue classification (>90%) and mainly confusion of pixels from healthy and tumor-associated stroma (Figure 5e). The ability to perform tissue classification on a low number of well-characterized metabolites outlines the potential to use the tested set-up for high-throughput, large-scale tissue classification studies as well as determination of tumor margins required for tumor resection surgery [29,30]. TQs are routinely used for clinical chemistry applications such as therapeutic drug monitoring, quantification of Vitamin D, and detection of metabolic deficiencies as part of newborn screenings. The robust mass analyzer technology allows for high scan rates on a low number of transitions, increasing throughput tremendously whilst significantly reducing bioinformatical needs for the data processing. The data acquisition in MRM mode ensures high quality data that do not need peak re-alignment or spectral re-calibration and can be directly used for multivariate analysis and tissue classification.

## 4. Discussion

High-resolution mass analyzers providing untargeted information are well suited for investigative study designs gathering a wealth of information in physiological, disease, and fundamental research. Utilizing this knowledge combined with the availability of in vivo diagnostics certified TQ mass spectrometers, TQs are routinely used for clinical screenings. The acquisition speed, instrumental robustness, and the small size of the resulting data files make the presented DESI-TQ setup the perfect fit for large-scale imaging studies with high sample throughput, allowing cost-effective data acquisition, storage, and processing.

Currently, untargeted data are primarily used to gain biological insights into the analyzed tissues, but they could also be used to identify metabolites suitable to be utilized in high-throughput imaging applications. Subjecting the data to univariate analysis allows the identification of metabolites with the highest predictive value for the different tissue types commonly found in biopsies. The top features with the highest significance can subsequently be identified in MS/MS experiments and the fragmentation pattern used to generate MRM tables for the routine imaging experiments on a DESI-TQ setup. As the TQ data are limited to the most discriminating metabolites, automated data processing pipelines could be established for routine analysis. These advantages leave room to envision the potential of semi- or fully automated setup-loading prepared slides, performing MSI analysis, staining the slides for histological evaluation, and presenting the optical scans alongside tissue classification results of the MSI—to allow MSI-guided histopathological evaluation of tissues. The guidance of the MSI data would providing pathologists with additional insight into the metabolic heterogeneity of the tissue alongside histologically stained tissue sections for easier interpretation and identification of tissue areas with complex histology, reducing workload and thus further increasing sample throughput.

Such an automated setup could equally be used for high-throughput elucidation of drug and drug metabolite distribution within biological samples alongside predefined assay panels to monitor for organ function and toxicity. This could be advantageous as first line in vivo screening for candidate selection in the pharmaceutical development process to evaluate compound properties, e.g., target engagement or blood–brain barrier penetration and help select the most promising candidates for further optimization. Lower throughput imaging modalities based on high mass resolving analyzer technology can then be utilized efficiently to study pharmacodynamic or toxicodynamic effects in-depth and in an untargeted way.

The accuracy of the data obtained by the TQ setup, in particular for the monitoring of endogenous metabolites, is dependent on the efforts taken to develop and validate the assay panels and the fragmentation properties of the metabolites. Selection and identification of metabolites for the development of such assay panels will be positioned at the back end of research studies as they are dependent on the insights obtained from scanning mass spectrometers or other sources. This makes the DESI-TQ setup dependent on the use of other instrument platforms in the same laboratory to generate the initial insight. However, predefined panels enable preferential utilization of the setup in settings with high sample throughput requirements, allowing to shift workload from more expensive, scanning instrument platforms to a sensitive, robust, and fast high-throughput imaging platform.

## Data Availability

All data relevant to the presented work is included in the main body of the manuscript or the supplementary information.

## Supplementary Materials

The following supporting information can be downloaded at: https://www.mdpi.com/article/10.3390/metabo13030377/s1, LC-MS based metabolite identification, Table S1: Instrument parameters for drug distribution study, Table S2: Instrument parameters for toxicity study, Table S3: Instrument parameters for tissue classification study, Table S4: MRM transitions for drug distribution study, Table S5: MRM transitions for toxicity study, Table S6: MRM transitions for tissue classification study, Figure S1: Realignment of the individual line scans, Figure S2: Ion images prior to realignment.
