# Supplementary material for: Targeted Desorption Electrospray Ionization Mass Spectrometry Imaging for Drug Distribution, Toxicity, and Tissue Classification Studies
Source: Metabolites. 2023 Mar 3;13(3):377. doi: 10.3390/metabo13030377 (PMC10060000; doi:10.3390/metabo13030377)
Supplement: Supplementary file 1 [file metabolites-13-00377-s001.zip › metabolites-2223536-supplementary.pdf]

Supplementary information: “Targeted desorption electrospray ionization mass spectrometry imaging for drug distribution, toxicity and tissue classification studies”

Andreas Dannhorn [1,2], Maria Luisa Doria [1], James McKenzie [1], Paolo Inglese [1], John G. Swales [2], Gregory Hamm [2], Nicole Strittmatter [2], Gareth Maglennon [3], Sadaf Ghaem-Maghami [1], Richard J. A. Goodwin [2,4] and Zoltan Takats [1,*]

[1] Department of Metabolism, Digestion and Reproduction, Faculty of Medicine, Imperial College London, London SW7 2AZ, UK
[2] Imaging and Data Analytics, Clinical Pharmacology and Safety Sciences, R&D, AstraZeneca, Cambridge CB4 0WG, UK
[3] Pathology, Clinical Pharmacology and Safety Sciences, R&D, AstraZeneca, Cambridge CB4 0WG, UK
[4] Institute of Infection, Immunity and Inflammation, College of Medical, Veterinary and Life Sciences, University of Glasgow, Glasgow G12 8QQ, UK
* Correspondence: z.takats@imperial.ac.uk

Table S1. Instrument parameters used for the drug distribution study

	TQ	QTof
Solvent	Methanol: Water (95:5)	Methanol: Water (95:5) containing 1 µg/ml raffinose for lock mass correction
Sprayer	Home-build Swagelok sprayer	Waters DESI-sprayhead
Flow rate	1.5 µL/min	0.75 µL/min
Gas pressure	7 bar	4 bar
Capillary voltage	4.5 kV	4.5 kV
Spatial resolution	50 µm	50 µm
Instrument Location	AstraZeneca	Imperial College London

Table S2. Instrument parameters used for the toxicity study of Polymyxin B induced kidney injury

	TQ
Solvent	Methanol: Water (95:5)
Sprayer	Home-build Swagelok sprayer
Flow rate	1.5 µL/min
Gas pressure	7 bar
Capillary voltage	4.5 kV
Spatial resolution	75 µm
Instrument Location	AstraZeneca

Table S3. Instrument parameters used for the tissue classification study:

	TQ
Solvent	Methanol: Water (95:5)
Sprayer	Waters DESI-sprayhead
Flow rate	2 µL/min
Gas pressure	6 bar
Capillary voltage	4 kV
Spatial resolution	100 µm
Instrument Location	Imperial College London

Table S4. MRM transitions used for the drug distribution study

Compound	Transition
Olanzapine	313.15>256.9
Erlotinib	394.18>278.09
Moxifloxacin	402.18>261.10
Terfenadine	472.32>436.30
Hydroxy-olanzapine	329.10>295.19
PC(34:1) $[M+K]^+$	798.54>739.47
PC (36:2) $[M+K]^+$	824.55>765.48
PC(38:4) $[M+K]^+$	844.53>785.46

Table S5. MRM transitions used for the toxicity study of Polymyxin B induced kidney injury

Compound	Transition
LPC 18:0 $[M+K]^+$	562.33>104.11
FA(20:4) $[M-H]^-$	303.23>285.22
FA(22:6) $[M-H]^-$	327.23>283.24
PE(18:2/20:4) $[M-H]^-$	762.51>303.23
PE(16:0/22:6) $[M-H]^-$	766.51>327.23
PE(18:0/20:4) $[M-H]^-$	766.54>303.23
PE(16:0/22:4) $[M-H]^-$	766.54>283.26

Table S6. MRM transitions used for the tissue classification study

Compound	Transition
SM(d34:1) $[M+H]^+$	703.6 > 184.0
SM(d34:1) $[M+Na]^+$	725.7 > 666.6
SM(d42:3) $[M+Na]^+$	809.7 > 750.6
PC(32:0) $[M+Na]^+$	756.6 > 697.5
PC(36:4) $[M+H]^+$	782.6 > 184.0
PC(34:1) $[M+K]^+$	798.6 > 739.5
PC(36:2) $[M+K]^+$	824.7 > 765.6

Re-alignment of the individual line scans:

The individual line scans showed an instrument dependent offset between start of the stage movement and the data acquisition. The offset results in horizontal shifts of the individual line scans in the compiled image. The offset is increasingly noticeable with increasing scan rates, resulting in significant distortion at 10 scans/s. To compensate for the shifts, the individual line scans were re-aligned to reduce the blurriness of the images in the drug distribution study. To perform the re-alignment, the tissues were identified by k-means clustering (2 cluster) performed on the whole dataset (Figure S2b). The first line scan of the tissue was manually selected, and subsequent lines were re-arranged by average smoothing of the tissue edge over 15 neighbouring lines (Figure S2c). The smoothed TIC image of the tissue section is displayed in Figure S2d.

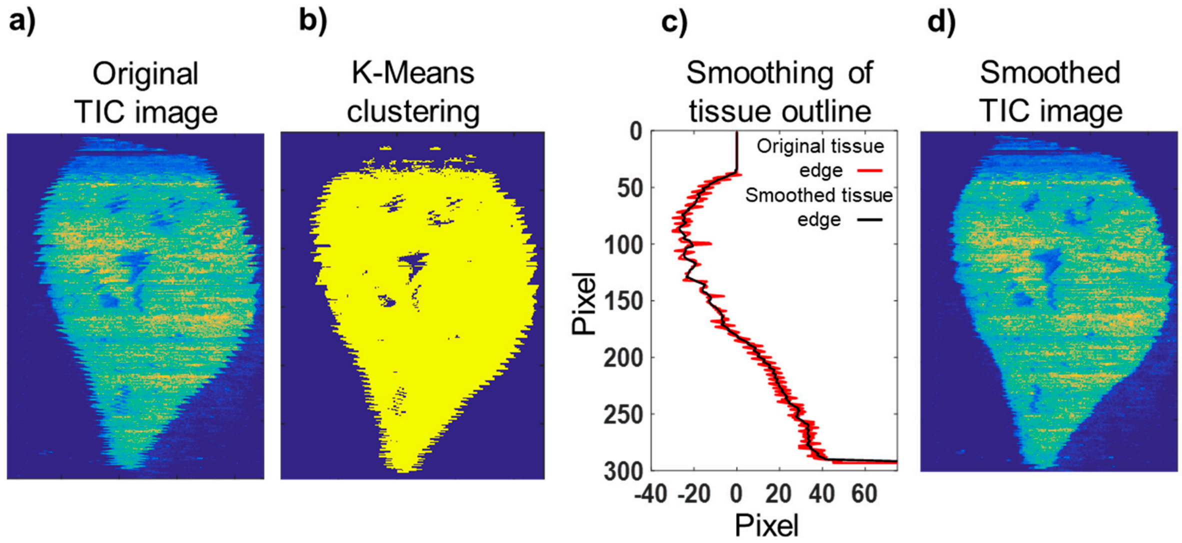

Figure S1. *a) Original total ion current (TIC) image of a rat liver section. b) K-means clustering (2 cluster) was used to distinguish between tissue (yellow) and background (blue). c) After manual selection of the first pixel, all subsequent lines were re-arranged by average smoothing over 15 adjacent line scans. d) The TIC image of the liver section after re-alignment.*

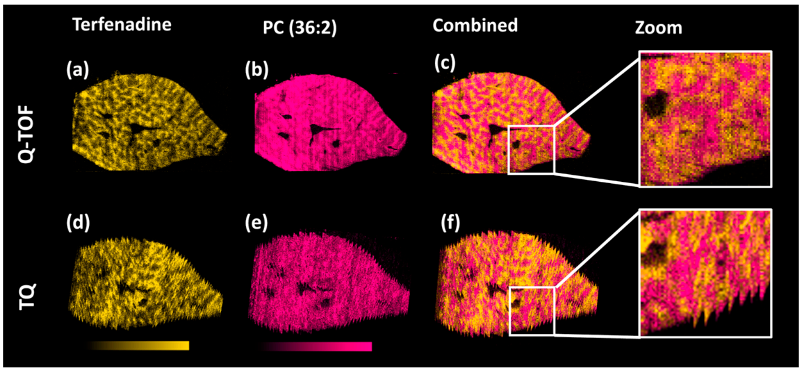

Figure S2*. Ion images for terfenadine (a,d) and endogenous lipid PC (36:2) (b,e) obtained by DESI-MSI performed on a Xevo G2-XS or a Xevo TQ-S. Figures c and f show the combined ion images of the drug and the endogenous lipid. The zoomed view displays the tissue edges. Images d-f are the original images prior re-alignment of the individual line scans.*
